# Chromosome-level genome and high nitrogen stress response of the widespread and ecologically important wetland plant *Typha angustifolia*

**DOI:** 10.3389/fpls.2023.1138498

**Published:** 2023-05-17

**Authors:** Yang Liao, Shuying Zhao, Wenda Zhang, Puguang Zhao, Bei Lu, Michael L. Moody, Ninghua Tan, Lingyun Chen

**Affiliations:** ^1^ School of Traditional Chinese Pharmacy, China Pharmaceutical University, Nanjing, China; ^2^ School of Environment and Ecology, Jiangsu Open University, Nanjing, China; ^3^ Key Laboratory of Plant Germplasm Enhancement and Specialty Agriculture, Wuhan Botanical Garden, Chinese Academy of Sciences, Wuhan, China; ^4^ Department of Biological Sciences, University of Texas at El Paso, El Paso, TX, United States

**Keywords:** genome sequencing, phytoremediation, high nitrogen stress, transcriptome, *Typha angustifolia*, eutrophication

## Abstract

*Typha angustifolia* L., known as narrowleaf cattail, is widely distributed in Eurasia but has been introduced to North America. *Typha angustifolia* is a semi-aquatic, wetland obligate plant that is widely distributed in Eurasia and North America. It is ecologically important for nutrient cycling in wetlands where it occurs and is used in phytoremediation and traditional medicine. In order to construct a high-quality genome for *Typha angustifolia* and investigate genes in response to high nitrogen stress, we carried out complete genome sequencing and high-nitrogen-stress experiments. We generated a chromosomal-level genome of *T. angustifolia*, which had 15 pseudochromosomes, a size of 207 Mb, and a contig N50 length of 13.57 Mb. Genome duplication analyses detected no recent whole-genome duplication (WGD) event for *T. angustifolia*. An analysis of gene family expansion and contraction showed that *T. angustifolia* gained 1,310 genes and lost 1,426 genes. High-nitrogen-stress experiments showed that a high nitrogen level had a significant inhibitory effect on root growth and differential gene expression analyses using 24 samples found 128 differentially expressed genes (DEGs) between the nitrogen-treated and control groups. DEGs in the roots and leaves were enriched in alanines, aspartate, and glutamate metabolism, nitrogen metabolism, photosynthesis, phenylpropanoid biosynthesis, plant-pathogen interaction, and mitogen−activated protein kinase pathways, among others. This study provides genomic data for a medicinal and ecologically important herb and lays a theoretical foundation for plant-assisted water pollution remediation.

## 1 Introduction

 More than 80% of the wastewater produced by human activities such as industry, agriculture, animal husbandry and daily life is directly discharged into rivers and oceans without treatment, causing serious water pollution (Wang et al., 2021; Lin et al., 2022). The main types of water pollutants include aerobic pollutants, heavy metals, pathogenic microorganisms, plant nutrients, etc. (Chang, 2006). Among these pollutants, nitrogen is one of the most common pollutants and has gained much attention (Byrnes et al., 2020). The increase of active nitrogen in the world not only pollutes the atmospheric environment, but also damages the aquatic ecosystem (Camargo and Alonso, 2006). Excessive nitrogen in soil and surface water increases the emission of greenhouse gas and aggravates global warming (Syakila and Kroeze, 2011). An overabundance of nitrogen in water leads to eutrophication and harmful algal blooms (Harding et al., 2019). Moreover, excessive nitrogen is harmful to humans. For example, a nitrate level of >10 mg/L in drinking water may cause diseases such as cancer and birth defects (Ward et al., 2018). Phytoremediation is a bioremediation technique that uses plants to remove pollutants from water by sorption, sedimentation, and decomposition (Ajibade et al., 2013). It is favored because of its many advantages, including low cost, low energy consumption, easy operation, purification of water, and stabilization of ecosystem structure (He et al., 2018).

*Typha angustifolia* L. (Typhaceae) is a large, perennial wetland plant with great adaptability that is native to Eurasia but has been introduced to North America (Sun and Simpson, 2011). *T. angustifolia* is also a famous medicinal herb. Its dried pollen, known as ‘*Pollen Typhae*’, is included in the ‘Chinese Pharmacopoeia’ (Chinese Pharmacopoeia Commission, 2020). Flavonoids are one of the main kinds of active components of Pollen Typhae. Pollen Typhae mainly includes flavonols (such as Typha neoglycoside, isorhamnetin-3-*O*-neohesperidin, isorhamnetin), flavanones (such as naringin), flavones and flavans (Chen P. et al., 2017; Ke et al., 2022). In leaves and pollens, quercetin-3, 3’-dimethyl ether, 3’-dimethyl eher-4’-*O*-*β*-D-glucoside can be also detected (Ghezal et al., 2017). No flavonoids was detected from the stem (Jamshaid et al., 2022). Whether the root contains flavonoids remain unknown. As far as we know, no study had reported the biological role of flavonoids in *Typha*. According to other studies, flavonoids take part in plant defense against pathogens, herbivores, and environmental stress (Treutter, 2005). As one of the most popular plants for the remediation of water pollution, *T. angustifolia* has shown great potential in the removal of heavy metals (Rai, 2008), mercury (Gomes et al., 2014), chromium, cadmium, lead, nickel, zinc, and copper (Bonanno and Cirelli, 2017; Sricoth et al., 2018) and was shown to improve water quality. *Typha angustifolia* was also the most effective in purifying water from septic effluents among 20 aquatic plants (Neralla et al., 1999). Its congeneric species, such as *T. domingensis*, are also used for treating wastewater (Di Luca et al., 2019).

Molecular biology techniques and transcriptomic strategies are important to explore for improving the effectiveness of phytoremediation (Kang, 2014). Understanding the genomic pathways and genes involved in uptake of contaminants will make it possible to make informed choices of plants and potential genomic modifications for phytoremediation. For example, the simultaneous overexpression of the *glutathione 1* gene and the *phytochelatin synthase 1* gene increased the tolerance of *Arabidopsis* to heavy metals, such as cadmium and arsenic (Guo et al., 2008). Likewise, overexpression of the *tryptophan synthase beta 1* gene in *Arabidopsis* and tomato plants enhanced their tolerance to excessive cadmium stress (Sanjaya et al., 2008). For aquatic plants to function in phytoremediation in eutrophic conditions they need to tolerate high nitrogen stress. Tolerance to low nitrogen stress has been well studied in crop plants, for example, Yan et al. (2021) investigated the gene expression patterns of two wheat cultivars under low nitrogen stress and found that the differences in their ability to tolerate low N stress were caused by different roles of calcium-related pathways. Leveraging transcriptomes to investigate excess nitrogen stress remains understudied, but some recent discoveries are providing insight. For example, Li et al. (2022) identified four transcription factors associated with excessive nitrogen stress in ryegrass. However, the genetic mechanisms of plant responses to high nitrogen stress remain poorly investigated.

With the development of DNA-sequencing technologies, *de novo* assembled transcriptomes have been widely used in differential gene expression (DGE) analyses to investigate new genes or genes associated with certain environmental stress responses or other external stimuli. For example, using DGE analyses, Zheng et al. (2022) investigated the molecular regulatory mechanism underlying the response to excess nitrogen in *Azolla*. Compared to a DGE analysis using a reference genome, a DGE analysis using a *de novo* assembled transcriptome without a reference genome can only identify a portion of the true differentially expressed genes (DEGs), which can result in a large number of false positives (Chen et al., 2019). Therefore, complete sequencing to assemble a reference genome is preferred when studying genetic mechanisms associated with environments or metabolites.

To provide a high-quality genome of *T. angustifolia* and to investigate genes in response to high nitrogen stress, we (1) carry out complete genome sequencing, assembly, and annotation for *T. angustifolia* and (2) examine growth patterns and DEGs when *T. angustifolia* is treated with excessive NH_4_Cl. Growth indicators, such as fresh weight, root length, and leaf length, are measured, and the differentially expressed genes in the roots and leaves are analyzed.

## 2 Materials and methods

### 2.1 Plant materials and sequencing

**Figure 1:**
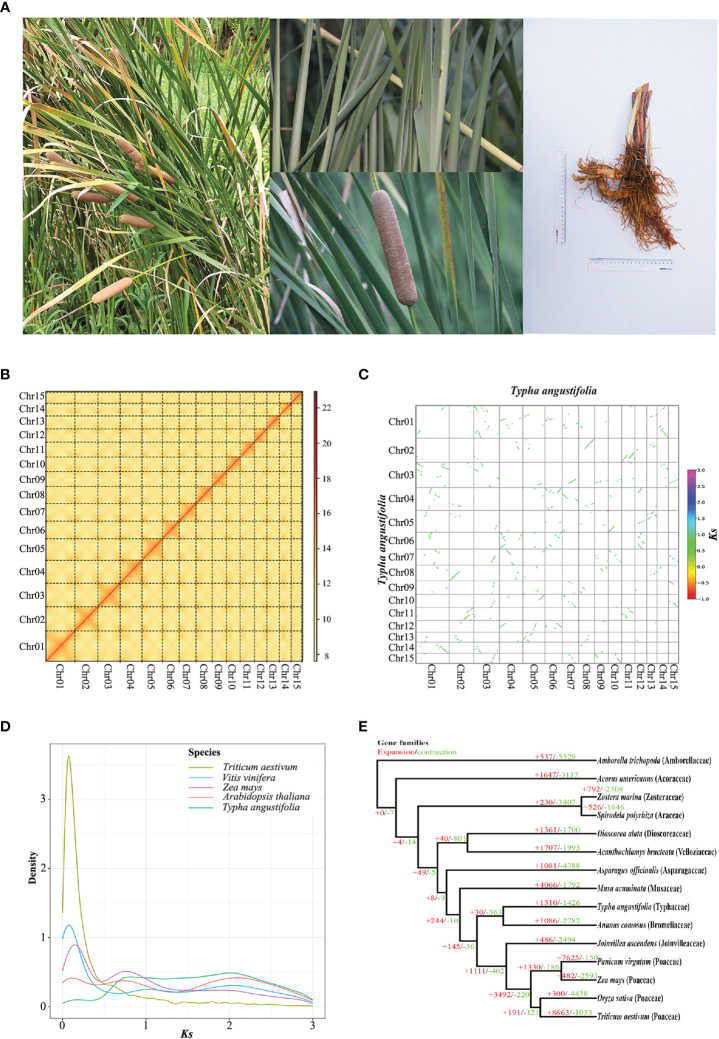
Genome evolutionary history of *T. angustifolia*. **(A)** Photos of *T. angustifolia* captured at the campus of China Pharmaceutical University in 2022. **(B)** Hi-C interaction heatmap for *T. angustifolia*. The diagonal lines of the rectangle indicate interactions within each chromosome. The color from light to dark indicates the intensity of interactions. **(C)** Scatter plot of *T. angustifolia* intraspecific synteny. **(D)** *Ks* distribution of *Arabidopsis thaliana*, *Triticum aestivum*, *T. angustifolia*, *Vitis vinifera*, and *Zea mays*. **(E)** Gene family expansion (+) and contraction (–) among 15 plant species.

*Typha angustifolia* was collected from Minghu, a natural lake on the campus of China Pharmaceutical University (CPU), Nanjing, China (**Figure 1A**). A voucher (no. LY220831211100CPU) was deposited in the herbarium of CPU. Leaves were used for genomic DNA extraction, while leaves and roots were used for total RNA extraction. Tissues used for PacBio HiFi sequencing and Illumina HiC sequencing were from the same seedling. Libraries were constructed according to the manufacturer’s standard protocol (Novogene, China). A HiFi SMRTbell library with a 20 kb insert size was sequenced using the PacBio Sequel II platform (Pacific Biosciences, USA). Illumina Novaseq sequencing, Hi-C sequencing and Transcriptome sequencing were performed using the Novaseq 6000 platform (Tianjin, China) with 150 bp paired-end reads.

### 2.2 Genome survey and genome assembly

 The genome size, heterozygosity, and repeat content of *T. angustifolia* were estimated using GenomeScope v2.0 with the following parameters: a k-mer length of 21 and a read length of 150 bp (Ranallo-Benavidez, 2020). The genomic information was also estimated with GCE v1.0.2 (Liu et al., 2013) using k-mer frequency distribution (k-mer = 21). *De novo* genome assembly was carried out using Nextdenovo v2.5.0 (https://github.com/Nextomics/NextDenovo ; accessed May 2022) with PacBio reads. Then, Hi-C sequencing reads were mapped to the assembly using ALLHiC v0.9.8 (Zhang et al., 2019). Next, JuiceBox v1.11.08 (Durand et al., 2016) was used to correct assembly errors, including the orientation, order, and internal mis-assembly of contigs. Finally, Benchmarking Universal Single-Copy Orthologs (BUSCO) v5.2.1 (Simao et al., 2015) and long terminal repeat (LTR) Assembly Index (LAI) v2.9.0 (Ou et al., 2018) were used to assess genome completeness and continuity.

### 2.3 Repeat annotation, gene prediction, and gene function annotation

 For repeat annotation, both homology-based and *de novo* approaches were used to search for TEs. EDTA v1.8.4 (Ou et al., 2019) and RepeatModeler v2.0.3 (Flynn et al., 2020) were used for *de novo* prediction. RepeatMasker v4.1.2-p1 (Tarailo-Graovac and Chen, 2009) with the Repbase database (updated: 20181026) (Bao et al., 2015) of known repeat sequences was used for homology-based prediction.

*De-novo*-based, homology-based, and RNA-seq-based approaches were used to identify coding genes (CDSs) in the genome assembly. *De novo* gene prediction was conducted using Augustus v3.3.3 (Stanke and Waack, 2003). Homology-based gene prediction was conducted using Genewise v2.4.1 (Birney et al., 2004), with protein sequences of five published genomes (*Oryza sativa*, *Zea mays*, *Ananas comosus*, *Elaeis guineensis*, and *Zingiber officinale*) as references. For the RNA-seq-based approach, Trimmomatic v0.39 (Bolger et al., 2014) was used for quality control of the RNA-seq raw data; then, HISAT2 v2.1.0 (Kim et al., 2019) was used to map reads to the *T. angustifolia* genome. Next, reliable intron information and optimal transcripts were obtained. Finally, TransDecoder v5.5.0 (Haas et al., 2013) was used to predict open reading frames (ORF) and gene models. The results from the three approaches were integrated using GETA v2.4.14 (https://github.com/chenlianfu/geta; accessed May 2022) to obtain the final protein-coding genes.

Protein-coding genes were annotated by aligning them with public databases using BLASTP (Camacho et al., 2009). The databases included the Gene Ontology (GO) (Blake et al., 2015), InterPro (Mitchell et al., 2019), NCBI non-redundant (NR) protein, Kyoto Encyclopedia of Genes and Genomes (KEGG) (Kanehisa and Goto, 2000), Swiss-Prot (Bairoch and Apweiler, 1996), and Pfam (Mistry et al., 2021) databases. Non-coding RNAs (ncRNAs) were annotated using Rfam v14.8 (Kalvari et al., 2021).

### 2.4 Whole-genome duplication

 Whole-genome duplication (WGD) has an important influence on plant evolution (Van de Peer et al., 2017). To identify WGD events in the *T. angustifolia* genome, WGDI v0.6.1 (Sun et al., 2022) was used. First, protein-coding genes of *T. angustifolia* were compared against themselves using BLASTP; second, synteny blocks were extracted; next, a synonymous substitutions per synonymous site (*Ks*) analysis was performed; and finally, the *Ks* distribution was plotted.

We also compared the WGD events among *T. angustifolia*, three monocotyledons (*Oryza sativa*, *Zea mays*, and *Triticum aestivum*), and one dicotyledon (*Arabidopsis thaliana*). The WGD events for each species were estimated using a *Ks*-based pipeline from Wang et al. (2019). In the pipeline, paralogous genes for each species were inferred using BLASTP; then, codeml in PAML (Yang, 2007) was used to infer the *Ks* value of each paralogous pair. Finally, the *Ks* distribution was plotted.

### 2.5 Gene family expansion and contraction

 Gene family expansion and contraction is an important factor affecting plant evolution. Coding sequences from 14 species (*T. angustifolia*, *Acanthochlamys bracteata*, *Acorus americanus*, *Ananas comosus*, *Asparagus officinalis*, *Dioscorea alata*, *Panicum virgatum*, *Joinvillea ascendens*, *Musa acuminata*, *Oryza sativa*, *Spirodela polyrhiza*, *Triticum aestivum*, *Zea mays*, and *Zostera marina*) of 11 families in 7 orders of monocotyledons, as well as *Amborella trichopoda* (outgroup), were used to assess gene family expansion and contraction. All data were accessed from Phytozome, except for that of *T. angustifolia* and *Acanthochlamys bracteata* (**Table S1**). First, OrthoFinder v2.5.4 (Emms and Kelly, 2019) was used to infer orthologous genes of the 15 species. Second, single-copy orthologous genes were extracted and aligned. A phylogenetic tree was constructed using a concatenated dataset formed from these genes with RAxML v8.2.12 (Stamatakis, 2014). Finally, CAFE4 v4.2.1 (Han et al., 2013) was used to infer gene family expansion and contraction.

### 2.6 Genome size and transposable elements

 Variation in plant genome size is mainly because of differing amounts of repeated sequences (Biémont, 2008). Transposable elements (TEs), a class of repeated sequences, comprise the majority of many eukaryotic genomes (Ou et al., 2019). TEs include two classes. Class I elements use RNA as an intermediate and move from one place to another *via* a “copy and paste” mechanism, while class II elements use DNA as an intermediate and moves from one place to another *via* a “cut and paste” mechanism (Ramakrishnan et al., 2022). Class I includes LTR retrotransposons (such as Copia and Gypsy), as well as those that lack LTRs (non-LTRs, such as the LINE element). Class II includes terminal inverted repeat (TIR) retrotransposons (such as CACTA and PIF Harbinger), and those that lack TIRs (non TIRs) (Ou et al., 2019). Limited evidence was provided to support the existence of an approximately linear relationship between TEs and genome size (Lee and Kim, 2014). *Typha* species have a small genome size, they are ideal models to study genome size variation and TE content.

To investigate the relationship between genome size and the types of TEs, genome sequences of 39 species were accessed from public databases (**Table S2**). The 39 species contained 26 monocotyledons and 13 dicotyledons. The largest genome was that of *Nicotiana tabacum* (3,584 Mb), and the smallest genome was that of *Genlisea aurea* (43 Mb). TEs in each genome were estimated using EDTA v1.8.4 (Ou et al., 2019). The correlation between genome size and the proportions of TEs was analyzed and plotted using Origin v2021.

### 2.7 Nitrogen stress experiments and differential gene expression analysis

 To investigate genes in response to high nitrogen stress, wet lab experiments were carried out. Healthy seedlings of *T. angustifolia* with similar heights were collected in March 2022 from Minghu (CPU campus) and grown in the greenhouse of the Medicinal Botanical Garden at CPU. Seedlings were washed and precultured in tap water for one month. Then, seedlings were precultured in modified Hoagland nutrient solution (**Table S3**; Tocquin et al., 2003; Zhu et al., 2022) for another month. Next, seedlings with similar heights and root lengths were divided into a control group (16 seedlings), treatment group 1 (500 mg/L NH_4_Cl; 12 seedlings), and treatment group 2 (900 mg/L NH_4_Cl; 12 seedlings). The fresh weight, root length, stem length, and leaf length of all 40 seedlings were measured at zero days, one month, and two months of stress (**Figure S1**). The solution was replaced every five days for the length of the experiment.

The most obvious phenotypic differences were observed between the control and treatment group 2, for which mean growth was inhibited after two months in all parts, in our preliminary experiments. Therefore, seedlings treated with 900 mg/L NH_4_Cl were used for RNA-seq. After three days of treatment, the roots and leaves were collected separately. Root and leaf tissues were also collected from the control group. In total, 6 replicates from a total of 24 samples were collected for RNA-seq for both the control group and the NH_4_Cl treatment group.

The pipeline of Chen et al. (2019) was used for differential gene expression (DGE) analysis. First, adapters and low-quality bases were filtered using Trimmomatic v0.39 (Bolger et al., 2014). Then, sequences from each sample were mapped to the CDSs of *T. angustifolia* using Salmon v1.3.0 (Patro et al., 2017). Tximport v1.6.0 (Soneson et al., 2015) was used to import Salmon outputs to DESeq2 v1.36 (Love et al., 2014). Finally, a DGE analysis was conducted using DESeq2. *P*-value < 0.05 and |log_2_(FoldChange)| > 1.5 were used as the criteria to quantify differential expression.

To annotate differentially expressed genes (DEGs), CDSs of DEGs were aligned with the TAIR database (Berardini et al., 2015). To explore the biological functions of the DEGs and determine whether they were associated with nitrogen stress and nitrogen metabolism, GO and KEGG enrichment analyses were performed using TBtools v1.1043 (Chen et al., 2020) for up- and downregulated genes in the roots and leaves separately.

## 3 Results and discussion

### 3.1 Genome sequencing, assembly, and annotation

 Sequencing for the genome survey generated 65.3 Gb (2 ×150 bp) of data. The survey indicated that the *T. angustifolia* genome was diploidy, with a size of 187 Mb, a heterozygosity of 0.365%, and a repetitive content of 9.8%. PacBio HiFi sequencing generated 10.26 Gb of data, with an average length of 15,029 bp and an N50 of 15,134 bp. Hi-C sequencing generated 66.8 Gb of data (2 ×150 bp). By leveraging the HiFi data, we generated an assembly with a size of 207 Mb in 45 contigs and an N50 of 12.74 Mb. By leveraging the Hi-C data, these contigs were assigned to 15 pseudochromosomes (**Figure 1B**). Previous studies have indicated that the haplotype of *T. angustifolia* has 15 chromosomes (Majovsky, 1976). The N50 of the pseudochromosomes was 13.57 Mb, and the mapping rate was 99.3%. Among the 1,614 conserved single-copy genes in BUSCO (version: embryophyta_odb10), 1,605 (98.6%) genes were completely retrieved, 6 (0.4%) were partially retrieved, and 3 (0.1%) were missing. Widanagama et al, 2022 reported a genome for *T. latifolia* that had 1,158 scaffolds, an N50 of 8.71 Mb, and a BUSCO score of 96.0%, therefore the quality of the *T. angustifolia* assembly is of higher quality than that of *T. latifolia* (Widanagama et al., 2022) (**Table 1**).

**Table 1:** Genome assembly and annotation statistics of two *Typha* genomes.

Genomic features	*Typha latifolia* (Widanagama et al., 2022)	*Typha angustifolia* (This study)
Sequencing		
Raw bases of Illumina (Gb)	138.6	65.3
Raw bases of Pacbio Sequel II (Gb)	86.8	10.26
Raw bases of Hic (Gb)	*	66.8
Raw bases of RNA-seq (Gb)	*	31.79
Assembly		
Genome size (Mb)	287	207
N50 of contigs (Mb)	8.71	12.74
N50 of scaffolds (Mb)	8.71	13.57
Number of contigs	1158	45
Number of scaffolds	1158	36
Complete BUSCOs (%)	96.03%	99.5%
Rate of GC (%)	38.07%	37.64%
Pseudochromosomes number	*	15
Pseudochromosomes size (Mb)	*	200
Annotation		
Number of predicted genes	27432	23289
Number of tRNAs	502	410
Number of rRNAs	2095	103
Number of miRNAs	214	203
Number of snRNAs	*	281
Repeat sequences (%)	43.84%	27.63%
Complete BUSCOs (%)	*	98.6%

* Data not available.

Gene annotation using GETA predicted 23,289 protein-coding genes, with an average length of 4,671 bp. On average, each predicted gene contained ca. 5.8 exons with a sequence length of 288 bp. A total of 21,555 out of 23,289 (92.56%) genes could be supported by the RNA-seq data. In addition, we identified non-coding RNA (ncRNA) genes, including 141 rRNA, 203 miRNA, and 792 other genes. The BUSCO results indicated that the annotation was 97.3% complete (1,591 of the 1,614 core genes were completely retrieved).

A total of 57.132 Mb of TEs occupying 27.60% of the *T. angustifolia* genome were annotated. The majority of the TEs were LTRs, accounting for 15.65% (3.32% Copia, 8.93% Gypsy, and 3.40% unknown) of the genome. Other TEs were non LTRs (0.51%), TIRs (7.3%), and non TIRs (1.45%).

### 3.2 Whole-genome duplication

 To explore WGD events in *T. angustifolia*, we conducted an intragenomic co-linearity analysis. The results indicated a 1:2 syntenic relationship (**Figure 1C**), implying that *T. angustifolia* might be suffered three events of WGD. Alternatively, at least two rounds of WGD could have occurred, as the *Ks* plot of *T. angustifolia* showed a peak at *Ks* ≈ 0.8 (**Figure 1D**) and a peak at *Ks* ≈ 2.0, suggesting that *T. angustifolia* might have experienced at least two WGD events. The peak at *Ks* ≈ 2.0 likely corresponds to an ancient WGD in monocots, such as the rho (*ρ*) WGD (Qiao et al., 2019). *Zea mays* and *T. angustifolia* shared a *Ks* peak at *Ks* ≈ 0.8, which might correspond to the sigma (σ) WGD that occurred in the Poales ancestor (McKain et al., 2016). Compared to *Zea mays*, *Arabidopsis*, *Triticum aestivum*, and *Vitis vinifera*, which have clear histories of WGD events (Middleton et al., 2014; Chen et al., 2019), *T. angustifolia* had no recent *Ks* peaks (*Ks* ≈ 0.1). Therefore, *T. angustifolia* had no recent WGD events. Our results support those of McKain et al. (2016), who investigated the WGD events in Poales using transcriptomes and genomes and found no recent WGD event for *Typha*.

### 3.3 Gene family expansion and contraction

 To investigate genes lost or gained in *T. angustifolia*, we estimated gene family expansion and contraction using CAFE4. The results (**Figure 1E**) showed that the genome of *T. angustifolia* lost 1,426 genes but gained 1,310 genes. Other Mononcot genomes were compared and the closest relative to *Typha* investigated, *Ananas comosus*, a relative of *T. angustifolia*, lost 2,782 genes but gained 1,086 genes. Others, including *Musa acuminata* (4,066 gained; 1,792 lost), *Triticum aestivum* (8,663 gained; 1,033 lost), and *Panicum virgatum* (7,625 gained; 150 lost) all had more gained genes than lost genes as well. The other nine monocot families investigate had more genes lost than gained. The most lost genes were found for *Amborella trichopoda* (lost 5,529 genes). *Typha angustifolia* was the only aquatic/semiaquatic taxon investigated that had more genes gained than lost.

We conducted GO and KEGG enrichment analyses for the lost genes and gained genes in *T. angustifolia* separately. The KEGG results show gained genes included enrichment in sinapoyltransferase activity, phosphoric diester hydrolase activity, acridone alkaloid biosynthesis, polyketide biosynthesis proteins, organismal systems, flavonoid biosynthesis, and environmental adaptation (**Figure S2**). Some of these genes can have a range of functions related to adaption of *T. angustifolia* in the aquatic environment. Phosphoric diester hydrolase activity is increased in phosporous limited plants and has been found to be particularly high for broad leaved emergent taxa (Rejmánková et al., 2011). Both Acridone alkaloid biosynthesis and flavonoid can act in plant defense. An aquatic environment is teeming with bacteria, viruses, and other microorganisms (Roux et al., 2016) as well as parasitic taxa, including trematodes, echinoderms, monogeneans, and crustaceans (Behringer et al., 2018). Acridone alkaloids have biological activities such as cytotoxic, antibacterial, and antiparasitic properties (Michael, 2017). Flavonoids exhibit multiple roles in plants in response to a wide range of environmental stimuli (Peer and Murphy, 2006; Roberts and Paul, 2006). Böttner et al. (2021) found that flavonoid glucosides promoted ecological adaptations in the aquatic plant *Spirodela polyrhiza* under different abiotic stresses, such as copper sulphate addition. Lee et al. (2022) found a direct link of increased flavonoid activity linked directly to predator defense in aquatic *Lemna* spp. The increase in flavonoids genes could be of particular interest for future work given its importance for *T. angustifolia* medicinal uses.

The lost genes for KEGG results included enrichmented in peptidase activity, endopeptidase activity, enzyme activator activity, diterpenoid biosynthesis, glutathione metabolism, and protein family metabolism (**Figure S2**). Our findings on the loss of diterpenoid biosynthesis among other terpenoid related genes are consistent with those of Chen et al. (2022), which revealed that, a large number of terpenoid genes were lost in aquatic monocots compared to terrestrial plants. The lost or gained genes in *T. angustifolia* could be related to its adaptation to aquatic and marsh environments, although further study is needed.

### 3.4 Genome size and transposable elements

**Figure 2:**
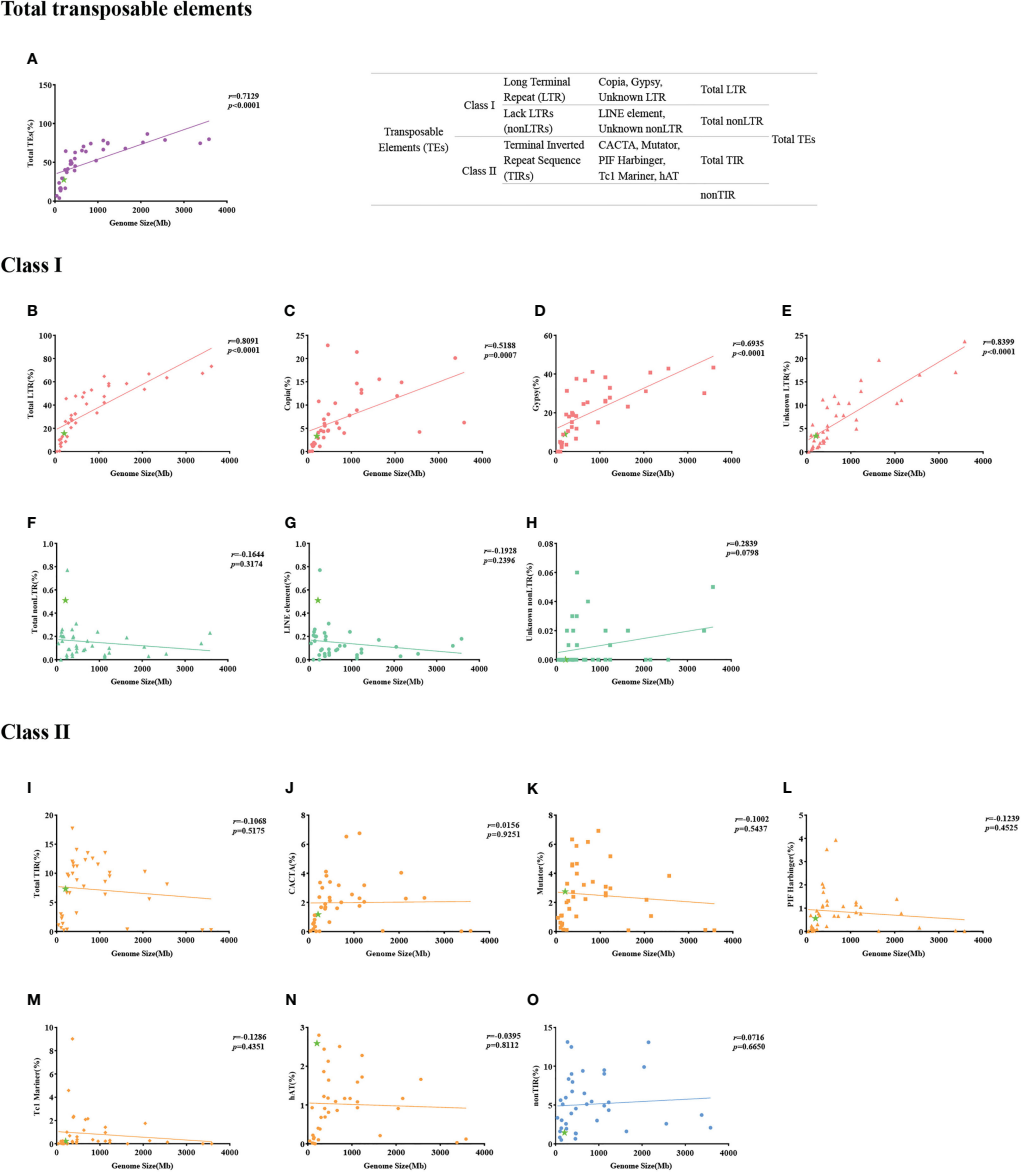
**(A–O)** Correlation between types of transposable elements and genome size for 39 angiosperm species.

 We used the genome sequences of 39 angiosperm species for a TE analysis. Our results showed that genome size correlated with the proportion of total TEs (**Figure 2A**), as well as the subclass LTRs (**Figure 2B**) of Copia (**Figure 2C**) and Gypsy (**Figure 2D**). For example, the correlation value between genome size and Gypsy was 0.6935, with a *p* value < 0.0001 (**Figure 2D**). However, no correlations with the proportion of total TIRs (**Figure 2I**) or their subclasses, such as CACTA (**Figure 2J**), Hat (**Figure 2N**), or Mutator (**Figure 2K**), were found (**Figure 2**). Zhang et al. (2020) found that the dynamic activity of Gypsy contributed to the vast diversity in genome size among Brassicaceae, but no correlation between genome size and proportion of TEs was found. Michael (2014) observed linear correlations between genome size and TEs, as well as with LTRs. Hawkins et al. (2009) found that smaller *Gossypium* genomes had a faster rate of LTR removal, and the genome size of *Gossypium* was correlated with Gypsy-like retrotransposons. Similar studies have confirmed that LTRs are removed from species with small genome sizes, such as *Utricularia gibba* (Ibarra-Laclette et al., 2013), *A. thaliana* (Devos et al., 2002), and *O. sativa* (Ma et al., 2004). Our results indicate that *T. angustifolia* had a small genome (207 Mb). The proportion of total TEs for *T. angustifolia* was 15.65%, a low proportion of TEs compared to species that have bigger genome sizes. The removal of LTRs could explain the linear correlation between genome size, the proportion of total TEs, and the small genome size of *T. angustifolia*. However, we realize that the small genome of *T. angustifolia* could also be attributed to the lack of a recent WGD event.

### 3.5 Phenotypic characteristics of *T. angustifolia* under high nitrogen treatment

**Figure 3:**
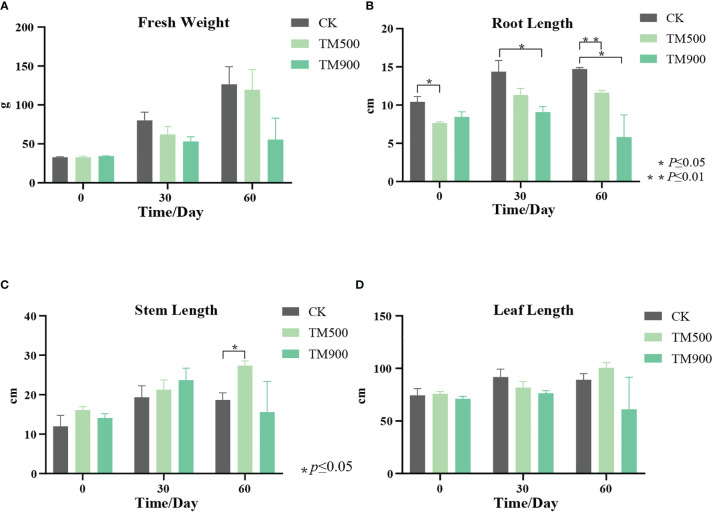
Growth of *T. angustifolia* under high nitrogen stress. **(A)** Fresh weight of *T. angustifolia*. **(B)** Root length of *T. angustifolia*. **(C)** Stem length of *T. angustifolia*. **(D)** Leaf length of *T. angustifolia*. CK: control group; TM500: 500 mg/L NH_4_Cl treatment group; TM900: 900 mg/L NH_4_Cl treatment group.

*Typha angustifolia* was grown in a control group (0 mg/L NH_4_Cl), a 500 mg/L NH_4_Cl treatment group, and a 900 mg/L NH_4_Cl treatment group for two months. Compared to the control group, the growth of the treated groups was inhibited at the end of two months, especially for the group treated with 900 mg/L NH_4_Cl (**Figure 3**; **Table S4**). The most obvious inhibition was found in the root length (**Figure 3B**). For example, the initial mean root length of the control group was 10.4 cm, and it increased to 14.7 cm after two months of growth. The initial mean root length of the 900 mg/L NH_4_Cl treatment group was 8.4 cm, but it decreased to 5.8 cm after two months of growth. Nitrogen availability is one of the main factors affecting plant growth and development (Kiba and Krapp, 2016). Root systems are the main organs to obtain nitrogen. The root growth could be induced by a low-nitrogen environment, but inhibited by a high-nitrogen environment (Xin et al., 2019). In a high NH_4_^+^ concentration, plants may accumulate excessive NH_4_^+^ in the cytosol, resulting in NH_4_^+^ toxicity. To alleviate NH_4_^+^ toxicity, the root cells have to excrete a large amount of NH_4_^+^, which leads to high energy cost, increased extra root respiration, and inhibited root growth (Britto et al., 2001).

Best (1980) found that low concentrations of ammonia and seven days of culturing stimulated the growth of the aquatic plant *Ceratophyllum demersum*, while the prolonged use (21 days) of ammonia caused toxic effects and inhibited growth. Similar to Best (1980), in our experiments, the roots of the 900 mg/L NH_4_Cl treatment group increased after one month of growth but decreased after two months of growth. *Typha* usually grows in ponds, marshes and wet banks of lakes and rivers. The nitrogen concentration of water in lake and pond, where *Typha angustifolia* is distributed, is at a range of 0.5–2.4 mg/L (Xu et al., 2014). However, the nitrogen concentration in severely polluted water could be as high as 500 mg/L (Clarke and Baldwin, 2002). In our experiment, we used a concentration of 900 mg/L of NH_4_Cl, which was much higher than that in natural habitats and most of the polluted water. Considering *T. angustifolia* has a great tolerance to nitrogen pollution, we used 900 mg/L of NH_4_Cl in our experiment. Moreover, no obvious morphological difference between the treated group (500 mg/L) and the control was observed in our preliminary experiments. Therefore, we used 900 mg/L of NH_4_Cl in our final experiments.

*Typha angustifolia* and its congeneric species were wildly used for the remediation of nitrogenate pollutants (Martín and Fernández, 1992; Ciria et al., 2005; Mufarrege et al., 2023). *T. angustifolia* was used as a carbon source of surface flow constructed wetland (SFCW) to improve the nitrogen removal rate (Wu et al., 2018). Cattail was planted in a constructed wetland to study its ability of treating polluted water (Gaballah et al., 2020). The results showed that the removal rate of total nitrogen was 94.7% and NH_3_-N removal was 99.9% (Gaballah et al., 2020).

The root length, stem length, leaf length and fresh weight of the treated group and control were measured. The results showed that the fresh weight and leaf length of the treated group were inhibited after two months of stress treatment, compared with the control group. The stem length was promoted for the treated group in the first month, but inhibited in the second month and after. The inhibition of leaf length is probably due to the regulation of the DEGs in leaves, such as xp11920 and xp18090 mentioned above. In addition, high nitrogen stress could also affect plant photosynthesis, plant fresh weight, and stem length (Mu and Chen, 2021).

No significant morphological differences were observed after 72 hours of stress treatment, but DEGs were detected. This phenomenon could be explained by two arguments: 1) The process from gene translation, mRNA modification, and protein translation to morphological changes may take some time (Ben-Ari et al., 2010). Therefore, the high nitrogen stress affected the gene expression after 72 hours of treatment, but had not affected the morphologies of *Typha angustifolia*. However, inhibition of root growth was observed after two months of high nitrogen treatment. 2) Alternatively, *Typha* spp. has a great tolerance to high nitrogen (Wang Y. et al., 2016). We could detect both DEGs and morphological changes if a higher concentration of nitrogen had been applied to our samples.

### 3.6 Genes in response to high nitrogen stress

**Figure 4:**
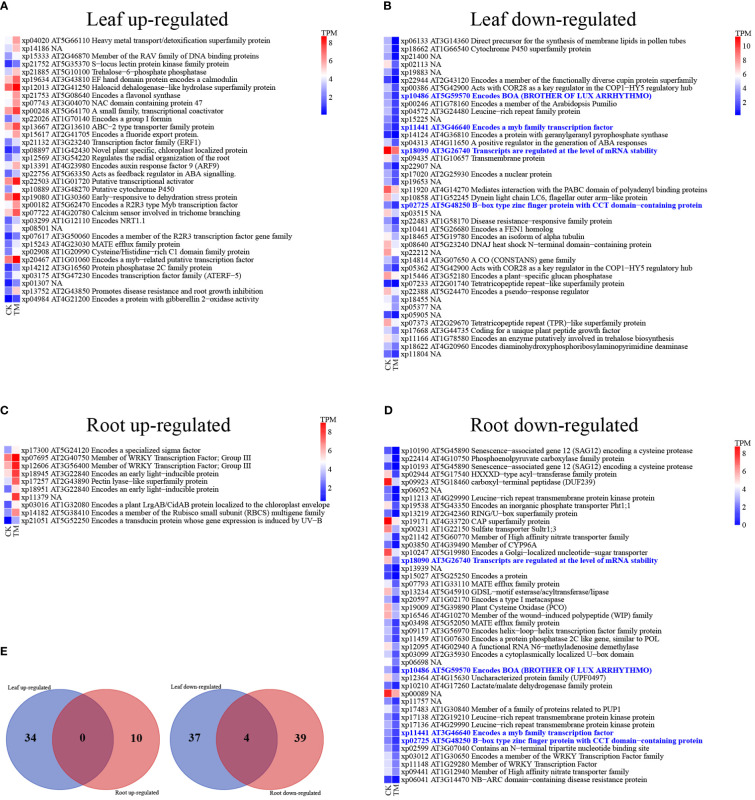
Comparative expression levels of differentially expressed genes in *T. angustifolia* after treatment with 900 mg/L NH_4_Cl for three days. **(A–D)** Gene expression values are presented as colors based on TPM scores for the control group (CK), and 900 mg/L NH_4_Cl treatment group (TM). Corresponding gene names were retrieved by aligning DEGs to the *Arabidopsis* genome using BLASTP. **(E)** The Venn diagrams show the numbers of DEGs specific to or common to the leaves and roots of *T. angustifolia*.

 To investigate gene responses to high nitrogen stress, we performed DGE analyses between the control group and the group treated with 900 mg/L NH_4_Cl (**Table S5**). The analyses recovered 128 DEGs (**Figure 4**; **Table S6**). This is a relatively small amount of DEGs recovered compared to other comparative transcriptomic studies examining ammonium stress using *de novo* assembled transcriptomes (Wang W. et al., 2016; Zhang et al., 2021). Wang et al. (2016) identified >14K unigenes that were differentially expressed under ammonium stress in the aquatic plant *Lemna minor*, while Zhang et al. (2021) found >30K for *Myriophyllum aquaticum*. The overall number of DEGs recovered in a study will reflect both treatment type and genome size of the organism, but also analytical method and experimental design. Using complete genome sequencing, rather than a *de novo* assembled transcriptome is expected to infer fewer false positives (Chen et al., 2019). In addition, the use of more replicates, six in our analyses, rather than the commonly used three replicates, can reduce false DEGs recovery (Li et al., 2020). Therefore, the methodology used here in part explains the comparatively low number of DEGs recovered and should provide high confidence in the accuracy of the result.

In plants, ammonium is transported by an ammonium transporter (AMT; Howitt and Udvardi, 2000). Moreover, glutamate dehydrogenase (GDH) plays a key role in maintaining the balance of nitrogen by regulating glutamate homeostasis (Grzechowiak et al., 2020). Differential expression of these and related genes would be expected in response to ammonium stress. For example, DEGs encoding AMT and GDH were downregulated and upregulated, respectively, in the aquatic fern *Azolla* under excess nitrogen (Zheng et al., 2022). DEGs encoding AMT were not found in our analyses. However, DEGs in the roots and leaves were upregulated for the ‘alanine, aspartate and glutamate metabolism’. Additionally, plant hormone signal transduction, ‘Valine, leucine and isoleucine degradation’, and photosynthesis were all recognized as upregulated in the KEGG analysis (**Figure S3**), similar to the ammonium stress response found for *Myriophyllum aquaticum* (Zhang et al., 2021).

We found 75 DEGs in the plant leaves, of which 34 were upregulated and 41 were downregulated (**Figure 4**). Similarly, more downregulated DEGs than upregulated DEGs were found in the aquatic duckweed (*Lemna minor*) under NH_4_^+^ toxicity (Wang W. et al., 2016). The upregulated genes included flavonol synthase, proteins promoting disease resistance, root growth inhibition, among others (**Figure 4**; **Figure S3**). We found 53 DEGs in the plant roots, of which 10 were upregulated and 43 were downregulated. It has been shown that, under high ammonia conditions, root cells undergo a futile transmembrane ammonia cycle resulting in a high energy cost (Li et al., 2014), which probably causes the upregulation of genes for root growth inhibition. Leaves and roots shared four downregulated genes but no upregulated genes (**Figure 4E**).

**Figure 5:**
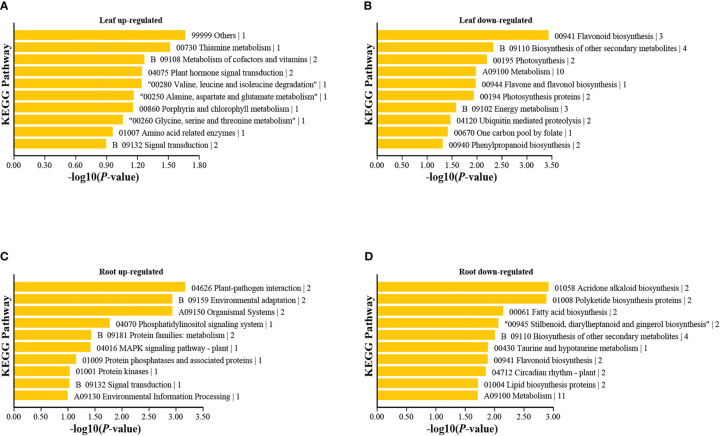
KEGG pathway enrichment analyses of differentially expressed genes in leaves and roots under high nitrogen stress. The 10 pathways with the smallest *p* values are presented for: **(A)** Leaf up-regulated, **(B)** Leaf down-regulated, **(C)** Root up-regulated, and **(D)** Root down-regulated. Other pathways are shown in **Figure S3**.

In the leaves, the GO and KEGG enrichment analyses showed that the upregulated DEGs were significantly enriched in response to external stimuli, thiamine metabolism, plant hormone signal transduction, signal transduction, among others (**Figure 5**; **Figure S3**). The downregulated DEGs were significantly enriched in the organic cyclic compound biosynthetic process, flavone and flavonol biosynthesis, photosynthesis proteins, nitrogen metabolism, cytochrome P450, phenylpropanoid biosynthesis, etc. (**Figure 5**). Nitrogen directly impacts the central plant metabolic ‘hub’—the phenylpropanoid biosynthesis pathway—from which important classes of molecules are formed, notably monolignols, flavonoids, and other types of polyphenols (Landi et al., 2019). For example, NH_4_^+^ toxicity caused the upregulation of phenylpropanoid biosynthesis in *L. minor* (Wang W. et al., 2016). However, our results showed that downregulated DEGs were enriched in phenylpropanoid and flavonol biosyntheses. This inconsistency could be explained by the fact that *T. angustifolia* has a higher content of flavonols (Chen P. et al., 2017), than *L. minor.* These two plants also have extreme phenotypic differences. *Typha* is a large emergent with aerial leaves and stems and sediment bound roots, whereas *L. minor* is a floating aquatic plant with a single small leaf, therefore energetic needs and gene regulation are likely different. To better understand gene regulation among aquatic plants more comparative experimental studies among growth forms and functional types will be required.

In the roots, upregulated DEGs were enriched in the plant-pathogen interaction, environmental adaptation, phosphatidylinositol signal transduction, and mitogen−activated protein kinase (MAPK) signal pathways, among others (**Figure 5C**). These results are similar to those of previous studies. For example, phosphatidylinositol signal transduction was reported to resist heat stress in *Pyropia haitanensis* (Wang et al., 2018), and the plant-pathogen interaction and MAPK pathways were associated with stress from polystyrene nanoplastics in wheat (Lian et al., 2022).

Some DEGs in roots were associated with plant growth, development, abiotic resistance, and biotic resistance. For example, xp10247, which is a homolog of the *GFT1* of *Arabidopsis* (At5g19980), encodes a Glogi-localized nucleotide-sugar transporter. *GFT1* is required for the normal growth and development of plants (Rautengarten et al., 2016). Xp19171, which is a homolog of the *ATCAPE3* gene in *Arabidopsis thaliana* (At4g33720), belongs to *CAP* (Cysteine-rich secretory proteins, Antigen 5, and Pathogenesis-related 1 protein) superfamily. This gene family includes plant pathogenesis-related proteins, which may act as an antifungal agent or participate in cell wall loosening (Lu et al., 2020). Xp09923, which belongs to the *Arabidopsis* carboxyl-terminal peptidase (DUF239, At5g18460), is a multifunctional regulator that regulates plant growth, stress, and auxin response (Ueda et al., 2008). Xp21051, which is a homolog of the *Arabidopsis EFO1* gene (At5g52250), encodes a transducer protein whose expression is induced by UV-B. Overexpression of this gene led to impaired plant growth and dwarfism (Gruber et al., 2010).

There are also some DEGs in the leaf in response to high nitrogen stress, such as xp11920 and xp18090, which belong to *PAM2* (At4g14270) motif and *CCL* (At3g26740) genes respectively. These genes have evolutionarily conserved and important functions including biosynthesis, transformation, and output (Albrecht and Lengauer, 2004; Lidder et al., 2005). Similarly, a high nitrogen stress in maize and ryegrass also affected the expression of genes in the development and growth stages (Li et al., 2022; Singh et al., 2022). These DEGs in leaf and roots not only participate in plant growth and development, but also have functions in responses to abiotic stress (Chen J. et al., 2017).

## 4 Conclusions

 This study resulted in a high-quality genome of an ecologically and medicinally important plant. We carried out high nitrogen stress experiments and differential gene expression analyses using a whole genome approach to investigated genomic response to high nitrogen stress. The results indicated that differentially expressed genes in roots and leaves were enriched in ‘alanine, aspartate and glutamate metabolism’, nitrogen metabolism, photosynthesis, phenylpropanoid biosynthesis, plant-pathogen interaction and mitogen-activated protein kinase pathways. Future studies on genetic mechanism of plant response to pollutants such as phosphorus and heavy metals and the development of cattails for phytoremediation could benefit from the genome in this study.

## Data availability statement

 The sequencing data was deposited in NCBI database under SRA accession numbers: PRJNA912339 (Survey, HiFi, and HiC sequencing data), PRJNA912578 (transcriptome data of 5 samples for genome assembly), PRJNA912863 (transcriptome data of 24 samples for high nitrogen stress).

## Author contributions

 YL & WZ: Data analyses & wet lab experiments. PZ: Wet lab experiments. SZ: Conceptualization and review. BL: Experimental and data analysis instruction. MM: Conceptualization and editing. NT: Conceptualization and review. LC: Conceptualization, review & editing. All authors contributed to the article and approved the submitted version.

## References

[B1] Ajibade F. O. Adeniran K. A. Egubuna C. K. (2013). Phytoremediation efficiencies of water hyacinth in removing heavy metals in domestic sewage (A case study of university of ilorin, Nigeria). IJES. 12 (2), 16–27. doi: 10.6084/m9.figshare.940965

[B2] Albrecht M. Lengauer T. (2004). Survey on the *PABC* recognition motif *PAM2* . Biochem. Biophys. Res. Commun. 316 (1), 129–138. doi: 10.1016/j.bbrc.2004.02.024 15003521

[B3] Bairoch A. Apweiler R. (1996). The SWISS-PROT protein sequence data bank and its new supplement TREMBL. Nucleic Acids Res. 24 (1), 21–25. doi: 10.1093/NAR/24.1.21 8594581 PMC145613

[B4] Bao W. Kojima K. K. Kohany O. (2015). Repbase update, a database of repetitive elements in eukaryotic genomes. Mob DNA. 6, 11. doi: 10.1186/s13100-015-0041-9 26045719 PMC4455052

[B5] Behringer D. C. Karvonen A. Bojko J. (2018). Parasite avoidance behaviours in aquatic environments. Phi. Trans. R. Soc. B. 373, 20170202. doi: 10.1098/rstb.2017.0202 PMC6000143 29866915

[B6] Ben-Ari Y. Brody Y. Kinor N. Mor A. Tsukamoto T. Spector D. L.. (2010). The life of an mRNA in space and time. J. Cell Sci. 123 (10), 1761–1774. doi: 10.1242/jcs.062638 20427315 PMC2864715

[B7] Berardini T. Z. Reiser L. Li D. Mezheritsky Y. Muller R. Strait E.. (2015). The arabidopsis information resource: making and mining the “gold standard”. annotated reference Plant Genome Genesis 53 (8), 474–485. doi: 10.1002/dvg.22877 26201819 PMC4545719

[B8] Best E. P. (1980). Effects of nitrogen on the growth and nitrogenous compounds of ceratophyllum demersum. Aquat Bot. 8, 197–206. doi: 10.1016/0304-3770(80)90051-0

[B9] Biémont C. (2008). Genome size evolution: within-species variation in genome size. Heredity 101, 297–298. doi: 10.1038/hdy.2008.80 18665185

[B10] Birney E. Clamp M. Durbin R. (2004). GeneWise and genomewise. Genome Res. 14 (5), 988–995. doi: 10.1101/gr.1865504 15123596 PMC479130

[B11] Blake J. A. Chan J. Kishore R. Sternberg P. W. Li Y. (2015). Gene ontology consortium: going forward. Nucleic Acids Res. 43, 1049–1056. doi: 10.1093/nar/gku1179 PMC4383973 25428369

[B12] Bolger A. M. Lohse M. Usadel B. (2014). Trimmomatic: a flexible trimmer for illumina sequence data. Bioinformatics 30 (15), 2114–2120. doi: 10.1093/bioinformatics/btu170 24695404 PMC4103590

[B13] Bonanno G. Cirelli G. L. (2017). Comparative analysis of element concentrations and translocation in three wetland congener plants: *Typha domingensis*, *Typha latifolia* and *Typha angustifolia* . Ecotox Environ. Safe. 143, 92–101. doi: 10.1016/j.ecoenv.2017.05.021 28525817

[B14] Böttner L. Grabe V. Gablenz S. Böhme N. Appenroth K. J. Gershenzon J.. (2021). Differential localization of flavonoid glucosides in an aquatic plant implicates different functions under abiotic stress. Plant Cell Environ. 44 (3), 900–914. doi: 10.1111/pce.13974 33300188

[B15] Britto D. T. Siddiqi M. Y. Glass A. D. Kronzucker H. J. (2001). Futile transmembrane NH_4_ ^+^ cycling: a cellular hypothesis to explain ammonium toxicity in plants. Proc. Natl. Acad. Sci. U S A. 98 (7), 4255–4258. doi: 10.1073/pnas.061034698 11274450 PMC31212

[B16] Byrnes D. K. Van Meter K. J. Basu N. B. (2020). Long-term shifts in U.S. nitrogen sources and sinks revealed by the new TREND-nitrogen data se–2017). Global Biogeochem Cy. 34, e2020GB006626. doi: 10.1029/2020GB006626

[B17] Camacho C. Coulouris G. Avagyan V. Ma N. Papadopoulos J. Bealer K.. (2009). BLAST+: architecture and applications. BMC Bioinf. 10, 421. doi: 10.1186/1471-2105-10-421 PMC2803857 20003500

[B18] Camargo J. A. Alonso A. (2006). Ecological and toxicological effects of inorganic nitrogen pollution in aquatic ecosystems: a global assessment. Environ. Int. 32 (6), 831–849. doi: 10.1016/j.envint.2006.05.002 16781774

[B19] Chang H. Q. (2006). Effectiveness and mechinesms of remediating eutrophic water body using aquatic plant-microbe integrated system (dissertation/master’s thesis) (Zhejiang Province, China: Zhejiang University).

[B20] Chen P. Cao Y. Bao B. Zhang L. Ding A. (2017). Antioxidant capacity of *Typha angustifolia* extracts and two active flavonoids. Pharm. Biol. 55 (1), 1283–1288. doi: 10.1080/13880209.2017.1300818 28274161 PMC7011981

[B21] Chen C. J. Chen H. Zhang Y. Thomas H. R. Frank M. H. He Y. H.. (2020). TBtools: an integrative toolkit developed for interactive analyses of big biological data. Mol. Plant 13 (8), 1194–1202. doi: 10.1016/j.molp.2020.06.009 32585190

[B22] Chen L. Y. Lu B. Morales-Briones D. F. Moody M. L. Liu F. Hu G. W.. (2022). Phylogenomic analyses of alismatales shed light into adaptations to aquatic environments. Mol. Biol. Evol. 39 (5), msac079. doi: 10.1093/molbev/msac079 35438770 PMC9070837

[B23] Chen L. Y. Morales-Briones D. F. Passow C. N. Yang Y. (2019). Performance of gene expression analyses using *de novo* assembled transcripts in polyploid species. Bioinformatics 35 (21), 4314–4320. doi: 10.1093/bioinformatics/btz620 31400193

[B24] Chen J. N. Nolan T. M. Ye H. X. Zhang M. C. Tong H. N. Xin P. Y.. (2017). *Arabidopsis WRKY46*, *WRKY54*, and *WRKY70* transcription factors are involved in brassinosteroid-regulated plant growth and drought responses. Plant Cell 29 (6), 1425–1439. doi: 10.1105/tpc.17.00364 28576847 PMC5502465

[B25] Chinese Pharmacopoeia Commission (2020). Pharmacopoeia of the people’s republic of China, vol. I (Beijing, China: China Medical Science Press), 368.

[B26] Ciria M. P. Solano M. L. Soriano P. (2005). Role of macrophyte *Typha latifolia* in a constructed wetland for wastewater treatment and assessment of its potential as a biomass fuel. Biosyst. Eng. 92 (4), 535–544. doi: 10.1016/j.biosystemseng.2005.08.007

[B27] Clarke E. Baldwin A. H. (2002). Responses of wetland plants to ammonia and water level. Ecol. Eng. 18 (3), 257–264. doi: 10.1016/S0925-8574(01)00080-5

[B28] Devos K. M. Brown J. K. Bennetzen J. L. (2002). Genome size reduction through illegitimate recombination counteracts genome expansion in *Arabidopsis* . Genome Res. 127, 1075–1079. doi: 10.1101/GR.132102 PMC186626 12097344

[B29] Di Luca G. A. Mufarrege M. M. Hadad H. R. Maine M. A. (2019). Nitrogen and phosphorus removal and *Typha domingensis* tolerance in a floating treatment wetland. Sci. Total Environ. 650 (Pt1), 233–240. doi: 10.1016/j.scitotenv.2018.09.042 30196224

[B30] Durand N. C. Shamim M. S. Machol I. Rao S. S. Huntley M. H. Lander E. S.. (2016). Juicer provides a one-click system for analyzing loop-resolution Hi-c experiments. Cell Syst. 3 (1), 95–98. doi: 10.1016/j.cels.2016.07.002 27467249 PMC5846465

[B31] Emms D. M. Kelly S. (2019). OrthoFinder: phylogenetic orthology inference for comparative genomics. Genome Biol. 20 (1), 238. doi: 10.1186/s13059-019-1832-y 31727128 PMC6857279

[B32] Flynn J. M. Hubley R. Goubert C. Rosen J. Clark A. G. Feschotte C.. (2020). RepeatModeler2 for automated genomic discovery of transposable element families. Proc. Natl. Acad. Sci. U S A. 117 (17), 9451–9457. doi: 10.1073/pnas.1921046117 32300014 PMC7196820

[B33] Gaballah M. S. Abdelwahab O. Barakat K. M. Aboagye D. (2020). A novel horizontal subsurface flow constructed wetland planted with *Typha angustifolia* for treatment of polluted water. Environ. Sci. pollut. Res. Int. 27 (22), 28449–28462. doi: 10.1007/s11356-020-08669-5 32418087

[B34] Ghezal N. Rinez A. Zribi I. Farooq M. Troisi L. Cannazza G.. (2017). Stimulatory effect on pea of *Typha angustifolia* l. extracts and their chemical composition. J. Plant Nutr. 40 (14), 1993–2005. doi: 10.1080/01904167.2017.1310893

[B35] Gomes M. V. de Souza R. R. Teles V. S. Araújo Mendes É. (2014). Phytoremediation of water contaminated with mercury using *Typha domingensis* in constructed wetland. Chemosphere 103, 228–233. doi: 10.1016/j.chemosphere.2013.11.071 24369743

[B36] Gruber H. Heijde M. Heller W. Albert A. Seidlitz H. K. Ulm R. (2010). Negative feedback regulation of UV-b-induced photomorphogenesis and stress acclimation in *Arabidopsis* . Proc. Natl. Acad. Sci. U S A. 107 (46), 20132–20137. doi: 10.1073/pnas.0914532107 21041653 PMC2993346

[B37] Grzechowiak M. Sliwiak J. Jaskolski M. Ruszkowski M. (2020). Structural studies of glutamate dehydrogenase (Isoform 1) from *Arabidopsis thaliana*, an important enzyme at the branch-point between carbon and nitrogen metabolism. Front. Plant Sci. 11. doi: 10.3389/fpls.2020.00754 PMC7326016 32655590

[B38] Guo J. Dai X. Xu W. Ma M. (2008). Overexpressing *GSH1* and *AsPCS1* simultaneously increases the tolerance and accumulation of cadmium and arsenic in *Arabidopsis thaliana* . Chemosphere 72 (7), 1020–1026. doi: 10.1016/j.chemosphere.2008.04.018 18504054

[B39] Haas B. J. Papanicolaou A. Yassour M. Grabherr M. Blood P. D. Bowden J.. (2013). *De novo* transcript sequence reconstruction from RNA-seq using the trinity platform for reference generation and analysis. Nat. Protoc. 8 (8), 1494–1512. doi: 10.1038/nprot.2013.084 23845962 PMC3875132

[B40] Han M. V. Thomas G. W. Lugo-Martinez J. Hahn M. W. (2013). Estimating gene gain and loss rates in the presence of error in genome assembly and annotation using CAFE3. Mol. Biol. Evol. 30 (8), 1987–1997. doi: 10.1093/molbev/mst100 23709260

[B41] Harding L. W. Mallonee M. E. Perry E. S. Miller W. D. Adolf J. E. Gallegos C. L.. (2019). Long-term trends, current status, and transitions of water quality in Chesapeake bay. Sci. Rep. 9, 6709. doi: 10.1038/s41598-019-43036-6 31040300 PMC6491606

[B42] Hawkins J. S. Proulx S. R. Rapp R. A. Wendel J. F. (2009). Rapid DNA loss as a counterbalance to genome expansion through retrotransposon proliferation in plants. Proc. Natl. Acad. Sci. U S A. 106 (42), 17811–17816. doi: 10.1073/pnas.0904339106 19815511 PMC2764891

[B43] He Y. Z. Xiang Y. J. Zhou Y. Y. Yang Y. Zhang J. C. Huang H. L.. (2018). Selenium contamination, consequences and remediation techniques in water and soils: a review. Environ. Res. 164, 288–301. doi: 10.1016/j.envres.2018.02.037 29554620

[B44] Howitt S. M. Udvardi M. K. (2000). Structure, function and regulation of ammonium transporters in plants. Biochim. Et Biophys. Acta 1465 (1–2), 152–170. doi: 10.1016/s0005-2736(00)00136-x 10748252

[B45] Ibarra-Laclette E. Lyons E. Hernández-Guzmán G. Pérez-Torres C. A. Carretero-Paulet L. Chang T. H.. (2013). Architecture and evolution of a minute plant genome. Nature 498 (7452), 94–98. doi: 10.1038/nature12132 23665961 PMC4972453

[B46] Jamshaid M. Rashid U. Butt Z. A. Munazir M. Qureshi R. (2022). Phytochemical analysis of methanolic extracts of *Elymus repens*, *Typha angustifolia* and *Caralluma edulis* . OARJBP. 6 (01), 081–088. doi: 10.53022/oarjbp.2022.6.1.0073

[B47] Kalvari I. Nawrocki E. P. Ontiveros-Palacios N. Argasinska J. Lamkiewicz K. Marz M.. (2021). Rfam 14: expanded coverage of metagenomic, viral and microRNA families. Nucleic Acids Res. 49 (D1), D192–D200. doi: 10.1093/nar/gkaa1047 33211869 PMC7779021

[B48] Kanehisa M. Goto S. (2000). KEGG: kyoto encyclopedia of genes and genomes. Nucleic Acids Res. 28 (1), 27–30. doi: 10.1093/nar/28.1.27 10592173 PMC102409

[B49] Kang J. W. (2014). Removing environmental organic pollutants with bioremediation and phytoremediation. Biotechnol. Lett. 36, 1129–1139. doi: 10.1007/s10529-014-1466-9 24563299

[B50] Ke J. H. An R. B. Cui E. J. Zheng C. J. (2022). Chemical constituents of the pollen of *Typha angustifolia* l. Biochem. Syst. Ecol. 104, 104460. doi: 10.1016/j.bse.2022.104460

[B51] Kiba T. Krapp A. (2016). Plant nitrogen acquisition under low availability: regulation of uptake and root architecture. Plant Cell Physiol. 57 (4), 707–714. doi: 10.1093/pcp/pcw052 27025887 PMC4836452

[B52] Kim D. Paggi J. M. Park C. Bennett C. Salzberg S. L. (2019). Graph-based genome alignment and genotyping with HISAT2 and HISAT-genotype. Nat. Biotechnol. 37 (8), 907–915. doi: 10.1038/s41587-019-0201-4 31375807 PMC7605509

[B53] Landi S. Berni R. Capasso G. Hausman J. F. Guerriero G. Esposito S. (2019). Impact of nitrogen nutrition on cannabis sativa: an update on the current knowledge and future prospects. Int. J. Mol. Sci. 20 (22), 5803. doi: 10.3390/ijms20225803 31752217 PMC6888403

[B54] Lee G. Choi H. Y. Joo Y. S. Kim S. G. (2022). Flavone-associated resistance of two *Lemna* species to duckweed weevil attack. Ecol. Evol. 12 (11), e9459. doi: 10.1002/ece3.9459 36415872 PMC9674451

[B55] Lee S. Kim N. (2014). Transposable elements and genome size variations in plants. Genomics Inform. 12, 87–97. doi: 10.5808/gi.2014.12.3.87 25317107 PMC4196380

[B56] Li B. Li G. Kronzucker H. J. Baluška F. Shi W. (2014). Ammonium stress in arabidopsis: signaling, genetic loci, and physiological targets. Trends Plant Sci. 19 (2), 107–114. doi: 10.1016/j.tplants.2013.09.004 24126103

[B57] Li Y. Wang M. Teng K. Dong D. Liu Z. Zhang T.. (2022). Transcriptome profiling revealed candidate genes, pathways and transcription factors related to nitrogen utilization and excessive nitrogen stress in perennial ryegrass. Sci. Rep. 12 (1), 3353. doi: 10.1038/s41598-022-07329-7 35233054 PMC8888628

[B58] Li Y. Yang H. Zhang H. Liu Y. Shang H. Zhao H.. (2020). Decode-seq: a practical approach to improve differential gene expression analysis. Genome Biol. 21 (1), 66. doi: 10.1186/s13059-020-01966-9 32200760 PMC7087377

[B59] Lian J. Liu W. Sun Y. Men S. Wu J. Zeb A.. (2022). Nanotoxicological effects and transcriptome mechanisms of wheat (*Triticum aestivum* l.) under stress of polystyrene nanoplastics. J. Hazard Mater. 423 (PtB), 127241. doi: 10.1016/j.jhazmat.2021.127241 34844359

[B60] Lidder P. Gutiérrez R. A. Salomé P. A. McClung C. R. Green P. J. (2005). Circadian control of messenger RNA stability. association with a sequence-specific messenger RNA decay pathway. Plant Physiol. 138 (4), 2374–2385. doi: 10.1104/pp.105.060368 16055688 PMC1183423

[B61] Lin L. Yang H. R. Xu X. (2022). Effects of water pollution on human health and disease heterogeneity: a review. Front. Env. Sci-Switz. 10. doi: 10.3389/fenvs.2022.880246

[B62] Liu B. H. Shi Y. J. Yuan J. Y. Hu X. S. Zhang H. Li N.. (2013). Estimation of genomic characteristics by analyzing k-mer frequency in *de novo* genome projects. arXiv: Genomics. doi: 10.48550/arXiv.1308.2012

[B63] Love M. I. Huber W. Anders S. (2014). Moderated estimation of fold change and dispersion for RNA-seq data with DESeq2. Genome Biol. 15 (12), 550. doi: 10.1186/s13059-014-0550-8 25516281 PMC4302049

[B64] Lu S. N. Wang J. Y. Chitsaz F. Derbyshire M. K. Geer R. C. Gonzales N. R.. (2020). CDD/SPARCLE: the conserved domain database in 2020. Nucleic Acids Res. 48 (D1), D265–D268. doi: 10.1093/nar/gkz991 31777944 PMC6943070

[B65] Ma J. Devos K. M. Bennetzen J. L. (2004). Analyses of LTR-retrotransposon structures reveal recent and rapid genomic DNA loss in rice. Genome Res. 14 (5), 860–869. doi: 10.1101/gr.1466204 15078861 PMC479113

[B66] Majovsky J. (1976). Index of chromosome numbers of slovakian flora part 5. Acta Facultatis Rerum Naturalium Universitatis Comenianae Botanica 25, 1–18.

[B67] Martín I. Fernández J. (1992). Nutrient dynamics and growth of a cattail crop (*Typha latifolia* l.) developed in an effluent with high eutrophic potential–application to wastewater purification systems. Bioresource Technol. 42 (1), 7–12. doi: 10.1016/0960-8524(92)90081-8

[B68] McKain M. R. Tang H. McNeal J. R. Ayyampalayam S. Davis J. I. dePamphilis C. W.. (2016). A phylogenomic assessment of ancient polyploidy and genome evolution across the poales. Genome Biol. Evol. 8 (4), 1150–1164. doi: 10.1093/gbe/evw060 26988252 PMC4860692

[B69] Michael T. P. (2014). Plant genome size variation: bloating and purging DNA. Brief Funct. Genomics 134, 308–317. doi: 10.1093/bfgp/elu005 24651721

[B70] Michael J. P. (2017). Acridone alkaloids. Alkaloids Chem. Biol. 78, 1–108. doi: 10.1016/bs.alkal.2017.06.001 28838426

[B71] Middleton C. P. Senerchia N. Stein N. Akhunov E. D. Keller B. Wicker T.. (2014). Sequencing of chloroplast genomes from wheat, barley, rye and their relatives provides a detailed insight into the evolution of the triticeae tribe. PloS One 9 (3), e85761. doi: 10.1371/journal.pone.0085761 24614886 PMC3948623

[B72] Mistry J. Chuguransky S. Williams L. Qureshi M. Salazar G. A. Sonnhammer E. L. L.. (2021). Pfam: the protein families database in 2021. Nucleic Acids Res. 49 (D1), D412–D419. doi: 10.1093/nar/gkaa913 33125078 PMC7779014

[B73] Mitchell A. L. Attwood T. K. Babbitt P. C. Bork P. Bridge A. Brown S. D.. (2019). InterPro in 2019: improving coverage, classification and access to protein sequence annotations. Nucleic Acids Res. 47 (D1), D351–D360. doi: 10.1093/nar/gky1100 30398656 PMC6323941

[B74] Mu X. H. Chen Y. L. (2021). The physiological response of photosynthesis to nitrogen deficiency. Plant Physiol. Biochem. 158, 76–82. doi: 10.1016/j.plaphy.2020.11.019 33296848

[B75] Mufarrege M. D. M. Di Luca G. A. Carreras Á.A. Hadad H. R. Maine M. A. Campagnoli M. A.. (2023). Response of *Typha domingensis* pers. in floating wetlands systems for the treatment of water polluted with phosphorus and nitrogen. Environ. Sci. Pollut. Res 30, 50582–50592. doi: 10.1007/s11356-023-25859-z 36800086

[B76] Neralla S. Weaver R. W. Varvel T. W. Lesikar B. J. (1999). Phytoremediation and on-site treatment of septic effluents in sub-surface flow constructed wetlands. Environ. Technol. 20, 1139–1146. doi: 10.1080/09593332008616911

[B77] Ou S. Chen J. Jiang N. (2018). Assessing genome assembly quality using the LTR assembly index (LAI). Nucleic Acids Res. 46 (21), e126. doi: 10.1093/nar/gky730 30107434 PMC6265445

[B78] Ou S. J. Su W. J. Liao Y. Chougule K. Agda J. R. A. Hellinga A. J.. (2019). Benchmarking transposable element annotation methods for creation of a streamlined, comprehensive pipeline. Genome Biol. 20 (1), 275. doi: 10.1186/s13059-019-1905-y 31843001 PMC6913007

[B79] Patro R. Duggal G. Love M. I. Irizarry R. A. Kingsford C. (2017). Salmon provides fast and bias-aware quantification of transcript expression. Nat. Methods 14 (4), 417–419. doi: 10.1038/nmeth.4197 28263959 PMC5600148

[B80] Peer W. A. Murphy A. S. (2006). “Flavonoids as signal molecules: targets of flavonoid action,” in The science of flavonoids. Ed. Grotewold E. (New York, NY: Springer), 239–268. doi: 10.1007/978-0-387-28822-2_9

[B81] Qiao X. Li Q. H. Yin H. Qi K. J. Li L. T. Wang R. Z.. (2019). Gene duplication and evolution in recurring polyploidization-diploidization cycles in plants. Genome Biol. 20 (1), 38. doi: 10.1186/s13059-019-1650-2 30791939 PMC6383267

[B82] Rai P. K. (2008). Heavy metal pollution in aquatic ecosystems and its phytoremediation using wetland plants: an ecosustainable approach. Int. J. Phytoremediat. 10 (2), 131–158. doi: 10.1080/15226510801913918 18709926

[B83] Ramakrishnan M. Satish L. Sharma A. Vinod K. K. Emamverdian A. Zhou M. B.. (2022). Transposable elements in plants: recent advancements, tools and prospects. Plant Mol. Biol. Rep. 40, 628–645. doi: 10.1007/s11105-022-01342-w

[B84] Ranallo-Benavidez T. R. Jaron K. S. Schatz M. C. (2020). GenomeScope 2.0 and smudgeplot for reference-free profiling of polyploid genomes. Nat. Commun. 11 (1), 1432. doi: 10.1038/s41467-020-14998-3 32188846 PMC7080791

[B85] Rautengarten C. Ebert B. Liu L. F. Stonebloom S. Smith-Moritz A. M. Pauly M.. (2016). The *Arabidopsis* golgi-localized GDP-_L_-fucose transporter is required for plant development. Nat. Commun. 7, 12119. doi: 10.1038/ncomms12119 27381418 PMC4935801

[B86] Rejmánková E. Sirová D. Carlson E. (2011). Patterns of activities of root phosphomonoesterase and phosphodiesterase in wetland plants as a function of macrophyte species and ambient phosphorus regime. New Phytol. 190 (4), 968–976. doi: 10.1111/j.1469-8137 21714183

[B87] Roberts M. R. Paul N. D. (2006). Seduced by the dark side: integrating molecular and ecological perspectives on the influence of light on plant defence against pests and pathogens. New Phytol. 170 (4), 677–699. doi: 10.1111/j.1469-8137.2006.01707.x 16684231

[B88] Roux S. Brum J. R. Dutilh B. E. Sunagawa S. Duhaime M. B. Loy A.. (2016). Ecogenomics and potential biogeochemical impacts of globally abundant ocean viruses. Nature 537 (7622), 689–693. doi: 10.1038/nature19366 27654921

[B89] Sanjaya Hsiao P. Y. Su R. C. Ko S. S. Tong C. G. Yang R. Y. (2008). Overexpression of *Arabidopsis thaliana tryptophan synthase beta 1* (*AtTSB1*) in *Arabidopsis* and tomato confers tolerance to cadmium stress. Plant Cell Environ. 31 (8), 1074–1085. doi: 10.1111/j.1365-3040.2008.01819.x 18419734

[B90] Simao F. A. Waterhouse R. M. Ioannidis P. Kriventseva E. V. Zdobnov E. M. (2015). BUSCO: assessing genome assembly and annotation completeness with single-copy orthologs. Bioinformatics 31, 3210–3212. doi: 10.1093/bioinformatics/btv351 26059717

[B91] Singh P. Kumar K. Jha A. K. Yadava P. Pal M. Rakshit S.. (2022). Global gene expression profiling under nitrogen stress identifies key genes involved in nitrogen stress adaptation in maize (*Zea mays* l.). Sci. Rep. 12, 4211. doi: 10.1038/s41598-022-07709-z 35273237 PMC8913646

[B92] Soneson C. Love M. I. Robinson M. D. (2015). Differential analyses for RNA-seq: transcript-level estimates improve gene-level inferences. F1000research. 4, 1521. doi: 10.12688/f1000research.7563.2 26925227 PMC4712774

[B93] Sricoth T. Meeinkuirt W. Pichtel J. Taeprayoon P. Saengwilai P. (2018). Synergistic phytoremediation of wastewater by two aquatic plants (*Typha angustifolia* and *Eichhornia crassipes*) and potential as biomass fuel. Environ. Sci. pollut. Res. Int. 25 (6), 5344–5358. doi: 10.1007/s11356-017-0813-5 29209971

[B94] Stamatakis A. (2014). RAxML version 8: a tool for phylogenetic analysis and post-analysis of large phylogenies. Bioinformatics 30 (9), 1312–1313. doi: 10.1093/bioinformatics/btu033 24451623 PMC3998144

[B95] Stanke M. Waack S. (2003). Gene prediction with a hidden Markov model and a new intron submodel. Bioinformatics 19 Suppl 2, ii215–ii225. doi: 10.1093/bioinformatics/btg1080 14534192

[B96] Sun P. C. Jiao B. B. Yang Y. Z. Shan L. X. Li T. Li X. N.. (2022). WGDI: a user-friendly toolkit for evolutionary analyses of whole-genome duplications and ancestral karyotypes. Mol. Plant 15 (12), 1841–1851. doi: 10.1016/j.molp.2022.10.018 36307977

[B97] Sun K. Simpson D. A. (2011). “Typha angustifolia,” in eFlora of China, vol. 23. Eds. Wu Z.Y. Peter H. R. (Beijing: Science Press; St. Louis: Missouri Botanical Garden Press), 161–162.

[B98] Syakila A. Kroeze C. (2011). The global nitrous oxide budget revisited. Greenhouse Gas Measurement Management. 1 (1), 17–26. doi: 10.3763/ghgmm.2010.0007

[B99] Tarailo-Graovac M. Chen N. S. (2009). Using RepeatMasker to identify repetitive elements in genomic sequences. Curr. Protoc. Bioinf. Chapter 4 4, 10. doi: 10.1002/0471250953.bi0410s25 19274634

[B100] Tocquin P. Corbesier L. Havelange A. Pieltain A. Kurtem E. Bernier G.. (2003). A novel high efficiency, low maintenance, hydroponic system for synchronous growth and flowering of *Arabidopsis thaliana* . BMC Plant Biol. 3, 2. doi: 10.1186/1471-2229-3-2 12556248 PMC150571

[B101] Treutter D. (2005). Significance of flavonoids in plant resistance and enhancement of their biosynthesis. Plant Biol. 7 (6), 581–591. doi: 10.1055/s-2005-873009 16388461

[B102] Ueda A. Li P. H. Feng Y. Vikram M. Kim S. Kang C. H.. (2008). The *Arabidopsis thaliana* carboxyl-terminal domain phosphatase-like 2 regulates plant growth, stress and auxin responses. Plant Mol. Biol. 67, 683–697. doi: 10.1007/s11103-008-9348-y 18506580

[B103] Van de Peer Y. Mizrachi E. Marchal K. (2017). The evolutionary significance of polyploidy. Nat. Rev. Genet. 18 (7), 411–424. doi: 10.1038/nrg.2017.26 28502977

[B104] Wang D. Hubacek K. Shan Y. Gerbens-Leenes W. Liu J. (2021). A review of water stress and water footprint accounting. Water-Sui. 13 (2), 201. doi: 10.3390/w13020201

[B105] Wang W. Li R. Zhu Q. Tang X. Zhao Q. (2016). Transcriptomic and physiological analysis of common duckweed *Lemna minor* responses to NH_4_ ^+^ toxicity. BMC Plant Biol. 16, 92. doi: 10.1186/s12870-016-0774-8 27091123 PMC4835947

[B106] Wang W. L. Lin Y. H. Teng F. Ji D. H. Xu Y. Chen C. S.. (2018). Comparative transcriptome analysis between heat-tolerant and sensitive *Pyropia haitanensis* strains in response to high temperature stress. Algal Res. 29, 104–112. doi: 10.1016/j.algal.2017.11.026

[B107] Wang Y. H. Wang J. F. Zhao X. X. Song X. S. Gong J. (2016). The inhibition and adaptability of four wetland plant species to high concentration of ammonia wastewater and nitrogen removal efficiency in constructed wetlands. Bioresource Technol. 202, 198–205. doi: 10.1016/j.biortech.2015.11.049 26708488

[B108] Wang N. Yang Y. Moore M. J. Brockington S. F. Walker J. F. Brown J. W.. (2019). Evolution of portulacineae marked by gene tree conflict and gene family expansion associated with adaptation to harsh environments. Mol. Biol. Evol. 36 (1), 112–126. doi: 10.1093/molbev/msy200 30371871

[B109] Ward M. H. Jones R. R. Brender J. D. de Kok T. M. Weyer P. J. Nolan B. T.. (2018). Drinking water nitrate and human health: an updated review. Int. J. Environ. Res. Public Health 15 (7), 1557. doi: 10.3390/ijerph15071557 30041450 PMC6068531

[B110] Widanagama S. D. Freeland J. R. Xu X. (2022). And Shafer, a Genome assembly, annotation, and comparative analysis of the *cattail typha latifolia* . B. A.G3 (Bethesda). 12 (2), jkab401. doi: 10.1093/g3journal/jkab401 PMC9210280 34871392

[B111] Wu S. Gao L. Gu J. Zhou W. Fan C. He S.. (2018). Enhancement of nitrogen removal *via* addition of *cattail* litter in surface flow constructed wetland. J. Clean Prod. 204, 205–211. doi: 10.1016/J.JCLEPRO.2018.09.036

[B112] Xin W. Zhang L. Zhang W. Z. Gao J. Yi J. Zhen X.. (2019). An integrated analysis of the rice transcriptome and metabolome reveals root growth regulation mechanisms in response to nitrogen availability. Int. J. Mol. Sci. 20 (23), 5893. doi: 10.3390/ijms20235893 31771277 PMC6928638

[B113] Xu Z. W. Zhang X. Y. Xie J. Yuan G. Tang X. Sun X.. (2014). Total nitrogen concentrations in surface water of typical agro- and forest ecosystems in chin-2009. PloS One 9 (3), e92850. doi: 10.1371/journal.pone.0092850 24667701 PMC3965473

[B114] Yan H. Shi H. Hu C. Luo M. Xu C. Wang S.. (2021). Transcriptome differences in response mechanisms to low-nitrogen stress in two wheat varieties. Int. J. Mol. Sci. 22 (22), 12278. doi: 10.3390/ijms222212278 34830160 PMC8622133

[B115] Yang Z. H. (2007). PAML4: phylogenetic analysis by maximum likelihood. Mol. Biol. Evol. 24 (8), 1586–1591. doi: 10.1093/molbev/msm088 17483113

[B116] Zhang Y. Li B. Liu F. Luo P. Wang Y. Liu D.. (2021). Transcriptomic and physiological analysis revealed the ammonium tolerance mechanisms of *Myriophyllum aquaticum* . Environ. Exp. Bot. 187, 104462. doi: 10.1016/j.envexpbot.2021.104462

[B117] Zhang S. J. Liu L. Yang R. Wang X. (2020). Genome size evolution mediated by gypsy retrotransposons in brassicaceae. Genom Proteom Bioinf. 18 (3), 321–332. doi: 10.1016/j.gpb.2018.07.009 PMC7801240 33137519

[B118] Zhang X. Zhang S. Zhao Q. Ming R. Tang H. (2019). Assembly of allele-aware, chromosomal-scale autopolyploid genomes based on Hi-c data. Nat. Plants. 5 (8), 833–845. doi: 10.1038/s41477-019-0487-8 31383970

[B119] Zheng X. Lin Z. Lu J. Ye R. Qu M. Wang J.. (2022). *De novo* transcriptome analysis reveals the molecular regulatory mechanism underlying the response to excess nitrogen in. Azolla Aquat Toxicol. spp, 248. doi: 10.1016/j.aquatox.2022.106202 35623198

[B120] Zhu S. J. Gui S. B. Xu W. Xiang P. Meng W. Zhu J.. (2022). Experimental research on the tolerance of four emergent plants under high nitrogen and phosphorus pollution conditions. China Rural Water Hydropower 0 (2), 34–38.

