# Supplementary material for: Chromosome-level genome and high nitrogen stress response of the widespread and ecologically important wetland plant Typha angustifolia
Source: Front Plant Sci. 2023 May 17;14:1138498. doi: 10.3389/fpls.2023.1138498 (PMC10230045; doi:10.3389/fpls.2023.1138498)
Supplement: Supplementary file 1 [file DataSheet_1.zip › Supplementary Material/Supplementary figures.doc]

**Figure S1 *Typha angustifolia* used in this study.**

**Figure S2 GO and KEGG enrichment of expanded and contracted genes in *Typha angustifolia*.**

**Figure S3 GO and KEGG enrichment of differentially expressed genes in *Typha angustifolia*.**
